# Increased Circulating Levels of Interleukin-6 Induce Perturbation in Redox-Regulated Signaling Cascades in Muscle of Dystrophic Mice

**DOI:** 10.1155/2017/1987218

**Published:** 2017-08-06

**Authors:** Laura Pelosi, Laura Forcina, Carmine Nicoletti, Bianca Maria Scicchitano, Antonio Musarò

**Affiliations:** ^1^DAHFMO-Unit of Histology and Medical Embryology, Sapienza University of Rome, Laboratory affiliated to Istituto Pasteur Italia-Fondazione Cenci Bolognetti, Rome, Italy; ^2^Institute of Histology and Embryology, School of Medicine, Catholic University of the Sacred Heart, Rome, Italy; ^3^Center for Life Nano Science@Sapienza, Istituto Italiano di Tecnologia, Rome, Italy

## Abstract

Duchenne muscular dystrophy (DMD) is an X-linked genetic disease in which dystrophin gene is mutated, resulting in dysfunctional or absent dystrophin protein. The pathology of dystrophic muscle includes degeneration, necrosis with inflammatory cell invasion, regeneration, and fibrous and fatty changes. Nevertheless, the mechanisms by which the absence of dystrophin leads to muscle degeneration remain to be fully elucidated. An imbalance between oxidant and antioxidant systems has been proposed as a secondary effect of DMD. However, the significance and precise extent of the perturbation in redox signaling cascades is poorly understood. We report that mdx dystrophic mice are able to activate a compensatory antioxidant response at the presymptomatic stage of the disease. In contrast, increased circulating levels of IL-6 perturb the redox signaling cascade, even prior to the necrotic stage, leading to severe features and progressive nature of muscular dystrophy.

## 1. Introduction

Duchenne muscular dystrophy (DMD) is an X-linked genetic disease caused by mutations in the dystrophin gene, leading to instability of the dystrophin-glycoprotein complex (DGC) with subsequent necrosis and fibrosis [1].

Although the genetic basis of DMD is known, the mechanisms by which the primary genetic defect leads to muscle wasting remain to be fully elucidated [2–4]. Among the potential factors involved in the pathogenesis of muscular dystrophy, oxidative stress [5, 6] might be responsible for the activation of degenerative processes and for the appearance and progress of pathologic changes in dystrophic muscles [4]. Nevertheless, the significance and precise role of excessive oxidant-related damage is poorly understood. The reactive oxygen species (ROS) are naturally and constantly formed inside of the organism, as a result of cell activity. It is plausible that under physiological conditions, skeletal muscle activates an endogenous program of antioxidant defense to maintain the ROS production at physiological levels [7]. Extreme or pathologic conditions generate much higher levels of ROS that overwhelm cellular antioxidant defenses, leading to protein carbonylation, DNA damage, and RNA oxidation. The excess of ROS production can also alter calcium handling, another pathogenic factor associated with muscular dystrophy, leading to the activation of proteolytic systems and muscle wasting [8, 9].

Different animal models of dystrophin deficiency are actively studied. The mdx mouse is the most widely used model for muscular dystrophy [2]. However, the mdx mouse presents some limitations, including the pathophysiology and clinical outcome criteria, compared to DMD patients, due to the fact that skeletal muscles of mdx mice undergo extensive necrosis only early in neonatal life. Thus, there is a greater imperative towards improving the validity of animal models and the design of preclinical experimental therapeutic approaches. We recently generated a more severe animal model, the mdx/IL6 mouse, that closely approximates the human disease and more faithfully recapitulates the disease progression in humans [10]. In particular, we observed that forced expression of IL-6 in the adult mdx mice, increased necrosis, sustained inflammatory response, and repeated cycles of muscle degeneration and regeneration, leading to exhaustion of satellite cells [10].

In this study, we analysed whether the exacerbated phenotype induced by increased circulating levels of IL-6 involves a perturbation in redox signaling cascade, even prior to the necrotic phase. We revealed, in mdx/IL6 mice, a progressive reduction of the Nrf2-dependent antioxidant compensatory mechanism, a severe phenotypic feature observed in human DMD patients [11].

## 2. Materials and Methods

### 2.1. Mice and Treatments

Animal models used in the current study are 2-, 4-, and 24-week-old wild-type C57Bl/6J mice and mdx (Jackson Laboratories) and mdx/IL6 [10] transgenic mice. moAb-IL6R treatment: mdx mice were injected subcutaneously, starting at 15 days of age, with the neutralizing monoclonal antibody MR16-1 (kindly provided by Chugai Pharmaceutical Co., Ltd.) [3, 12] to the murine IL-6 receptor (moAb-IL6R) at a dose of 100 *μ*g/g of body weight and then twice a week with 20 *μ*g/g (for a total of 5 doses) in PBS [3]. Mice were sacrificed at 4 weeks of age. Animals were maintained according to the institutional guidelines of the animal facility of the unit of Histology and Medical Embryology. All the animal experiments were approved by the ethics committee of Sapienza University of Rome-Unit of Histology and Medical Embryology and were performed in accordance with the current version of the Italian law on the protection of animals.

### 2.2. Protein Extraction and Western Blot Analysis

Diaphragm muscles were isolated from 2-, 4-, and 24-week-old mdx and mdx/IL6 mice and immediately frozen in liquid nitrogen. Each sample (liquid nitrogen powdered diaphragm muscles) was homogenized in protein lysis buffer [Tris-HCl, pH 7.5/20 mM, EDTA/2 mM, EGTA/2 mM, sucrose/250 mM, DTT/5 mM, Triton-X/0.1%, PMSF/1 mM, NaF/10 mM, SOV4/0.2 mM, cocktail protease inhibitors/1X (Sigma Aldrich)]. Western blotting analysis was performed using 70 *μ*g of protein extracts, and filters were blotted with antibodies against gp91phox (BD Transduction Laboratories), Nrf2 (Santa Cruz Biotechnology), NF*κ*B p65 (ser536; Cell Signaling), NF*κ*B (Cell Signaling), *α*-tubulin (Sigma Aldrich), *β*-tubulin (Cell Signaling), Glu-tubulin (detyrosinated *α*-tubulin; Abcam), and GAPDH (Santa Cruz Biotechnology). Signals derived from appropriate HRP-conjugated secondary antibodies (Bethyl Laboratories) were captured by Chemi DocTM XRS 2015 (Bio-Rad Laboratories), and densitometric analysis was performed using Image Lab software (version 5.2.1; Bio-Rad Laboratories^©^).

### 2.3. RNA Extraction and Quantitative Real-Time PCR Analysis

The total mRNA of 2-, 4-, and 24-week-old wild-type, mdx, and mdx/IL6 mice was extracted from liquid nitrogen powdered diaphragm muscle in TRI Reagent (Sigma-Aldrich) using Tyssue Lyser (Qiagen). To synthesize double-stranded cDNA, 1 *μ*g of each RNA sample was reverse transcribed using the QuantiTect Reverse Transcription kit (Qiagen). For the analysis of IL-6, TNF*α*, IL-1*β*, IL-10, and IL6r*α*, cDNA from each sample was preamplified using the TaqMan PreAmp Master Mix (Applied Biosystem) according to the manufacturer's protocol. Quantitative real-time PCR was performed, with or without preamplification procedure, on ABI PRISM 7500 SDS (Applied Biosystem) using specific 6-carboxyfluorescein (FAM)-labeled TaqMan assays for SIRT1, Utrn, SOD1, SOD2, CAT-1, Gpx1, GCLC, GCLM, NQO1, HO-1, IL-6, TNF*α*, IL-1*β*, IL-10, IL6r*α*, and Hprt as housekeeping genes (Applied Biosystem). Data were analysed using the 2-DDCt method and reported as mean fold change in gene expression relative to wild type.

### 2.4. Dihydroethidium Staining and Confocal Analysis for ROS Detection

Muscles from wild-type, mdx, mdx/IL-6, and mdx-treated mice with the neutralizing monoclonal antibody to the IL-6 receptor (moAb-IL6R) were embedded in tissue freezing medium and snap frozen in nitrogen-cooled isopentane. Muscle frozen sections (30 *μ*m) were incubated with 5 *μ*M dihydroethidium (DHE) (Molecular Probes; #D23107) in PBS at 37°C for 30 min [13] and analysed at confocal microscopy (Laser-Scanning TCS SP2; Leica) to reveal ROS production. DHE fluorescence was analysed with LAS AF Lite software, measuring the total intensity of DHE fluorescence, which represents the full amount of fluorescence held within the entire z-axis of the series. 60 optical sections/genotype for three separate experiments were analysed.

### 2.5. Data and Statistical Analysis

Statistical analysis was performed using the GraphPad Prism software (San Diego, CA, USA). Statistically significant differences among groups were assessed using one-way ANOVA with a Bonferroni's posttest or Dunn's posttest and between pairs with Mann–Whitney test or Student's *t*-test for normally distributed variables. All data are presented as mean ± SEM; *p* < 0.05 is considered statistically significant. Sample size was predetermined based on the variability observed in preliminary and similar experiments. All experiments requiring animal models were subjected to randomization based on litter.

## 3. Results

### 3.1. Increased Levels of IL-6 Cytokine Contribute to Enhance Markers of ROS Production in mdx Mouse Model

The progress of the dystropathology in the mdx mouse model has been extensively described. A prenecrotic stage, characterized by normal myofibres with peripheral nuclei, intact sarcolemma, and nonfragmented sarcoplasm, is observed within the first 21 days of age. Necrosis peaks by 25-26 days and then significantly decreases to stabilize by 8 weeks of age to a relatively low level of active damage [2]. Among the secondary processes that accompany muscle degeneration, the infiltration of inflammatory cells reflects the immune response to tissue damage.

**Figure 1:**
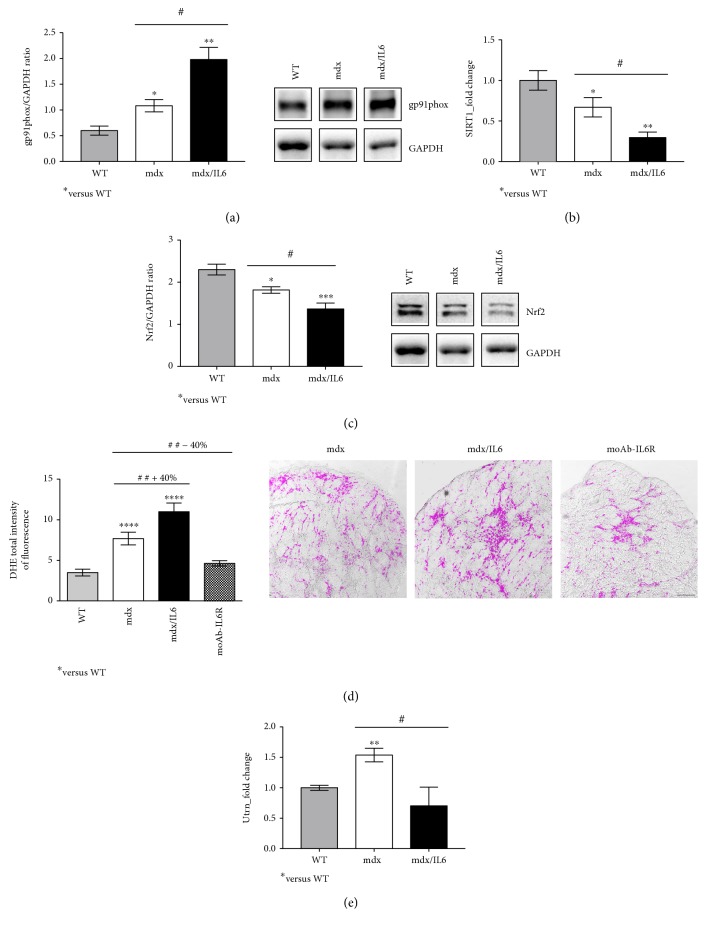
Analysis of redox-regulating signaling in the diaphragm muscle of 2-week-old dystrophic mice. Western blot analysis (right panels show representative images) for the expression of gp91phox (a) and Nrf2 (c) proteins in 2-week-old wild-type (WT), mdx, and mdx/IL6 mice. Values represent mean ± SEM; *n* = 3 to 6 mice per group. ^∗^*p* < 0.05, ^∗∗^*p* < 0.005, and ^∗∗∗^*p* < 0.0005 compared to WT mice; ^#^*p* < 0.05 between mdx and mdx/IL6 littermates by ANOVA. Real-time PCR analysis performed on diaphragm muscles from WT, mdx, and mdx/IL6 mice at 2 weeks of age for the expression of SIRT1 (b) and utrophin (Utrn) (e). Values are reported as fold change in expression and represent mean ± SEM; *n* = 4 to 12 per group. ^∗^*p* < 0.05, ^∗∗^*p* < 0.005 compared to WT mice; ^#^*p* < 0.05 (by ANOVA). (d) Blockade of IL-6 receptor by moAb-IL6R neutralizing antibody reduces the amount of DHE-derived fluorescence in treated mdx compared to untreated mdx mice. Graphs (left panel) show the quantification of DHE total intensity in the muscles of indicated genotypes. The right panel shows representative images of DHE staining from the muscle sections of 4-week-old mdx/IL-6 and moAb-IL6R-treated and untreated mdx mice. Scale bar, 100 *μ*m. Values represent mean ± SEM; *n* = 3 independent experiments. ^∗∗∗∗^*p* < 0.0001, ^##^*p* < 0.005 using ANOVA. In (a) and (c), the lanes were run on the same gel but were not contiguous.

To verify whether oxidative stress is a direct consequence of necrosis and inflammation or whether it can be induced in the prenecrotic stage, we analysed relevant markers of the redox signaling in the muscle of 2-week-old mdx mice. We found a strong upregulation of gp91phox (NOX2), the main source of ROS production [14, 15], in the diaphragm of 2-week-old mdx mice compared to wild-type littermates (Figure 1(a)), suggesting the presence of pro-oxidant conditions during the early disease stages. This was supported by a significant reduction in SIRT1 and Nrf2 expressions (Figures 1(b) and 1(c)), which are important mediators that promote the antioxidant response by activating several antioxidant enzymes [16–20].

It has been reported that, although with the absence of significant infiltration of mononuclear inflammatory cells within the dystrophic muscle, DMD patients display an increase in the plasma levels of proinflammatory cytokines at the presymptomatic stage of the disease [21]. Thus, the release of specific proinflammatory cytokines can stimulate ROS production, enhancing cellular damage in DMD [22]. To support this evidence, we analysed relevant markers of the oxidant and antioxidant signaling in the muscle of mdx/IL6 mice [10] at the prenecrotic stage. Increased IL-6 plasma levels induced a strong upregulation of gp91phox expression (Figure 1(a)) accompanied by a significant downregulation of SIRT1 and Nrf2 expressions (Figures 1(b) and 1(c)) in 2-week-old mdx/IL6 mice, compared to both wild-type and mdx littermates. This suggests that IL-6 promotes a perturbation of redox status in dystrophic muscles, modulating relevant factors of the redox signaling even in the prenecrotic phase.

To casually link IL-6 overexpression with ROS production in DMD pathology, we treated 2-week-old mdx mice with the neutralizing monoclonal antibody to the IL-6 receptor (moAb-IL6R), as previously described [3]. Fifteen days after the treatment, we analysed the ROS-sensitive dye DHE in the muscle of untreated and moAb-IL6R-treated mdx mice.  Figure 1(d) shows a strong reduction of DHE fluorescence in moAb-IL6R-treated mdx compared to untreated mdx mice, indicating that IL-6 blockade prevents excessive ROS production in dystrophic muscles. Notably, the muscle of mdx/IL6 mice displayed a significant increase of DHE fluorescence compared to that of control mdx and moAb-IL6R-treated mdx littermates (Figure 1(d)), supporting the role of IL-6 in the induction of pathologic changes observed at prenecrotic stage.

Overexpression of utrophin (Utrn) is able to counteract the lack of dystrophin protecting the sarcolemma integrity in the dystrophic muscle [23, 24]. Thus, we evaluated the levels of Utrn mRNA in the diaphragm of 2-week-old wild-type, mdx, and mdx/IL6 mice and we found a significant upregulation of its expression in mdx mice, compared to both wild-type and mdx/IL6 mice (Figure 1(e)). Interestingly, IL-6 overexpression reduced Utrn expression compared to mdx littermates, further supporting the evidence that the mdx/IL6 mouse model closely approximates the human disease and more faithfully recapitulates the disease progression in humans.

### 3.2. Analysis of Nrf2 Antioxidant Genes in Muscle of Dystrophic Mice at Prenecrotic Stage

**Figure 2:**
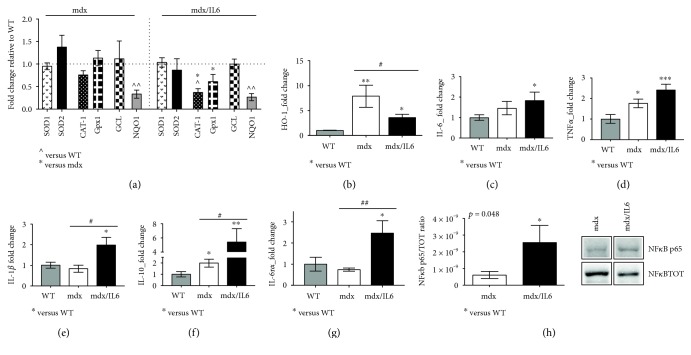
Nrf2 antioxidant genes are differently regulated in the prenecrotic dystrophic muscle. (a) Real-time PCR analysis of Nrf2-dependent genes (SOD1, SOD2, CAT-1, Gpx1, GCL, and NQO1) performed on the diaphragm muscle of 2-week-old WT, mdx, and mdx/IL6 mice. Values are reported as fold change in expression relative to the calibrator (WT, horizontal dot line) and represent mean ± SEM; *n* = 3 to 5 per group. *p* value by unpaired statistical tests. ^^^*p* < 0.05, ^^^^*p* < 0.005. Analysis of HO-1 mRNA (b), IL-6 mRNA (c), TNF*α* mRNA (d), IL-1*β* mRNA (e), IL-10 mRNA (f), and IL6r*α* mRNA (g) expressions in the diaphragm of 2-week-old WT, mdx, and mdx/IL6 mice. Data are presented as the mean ± SEM; *n* = 3 to 6 mice per group. ^∗^*p* < 0.05, ^∗∗^*p* < 0.005, and ^∗∗∗^*p* < 0.0005 compared to WT mice; ^#^*p* < 0.05, ^##^*p* < 0.005 between mdx and mdx/IL6 littermates (by ANOVA). (h) Densitometric analysis (left panel) and representative western blot (right panel) for NF*κ*B active (NF*κ*B p65) and total (NF*κ*B TOT) protein expression in the diaphragm muscle of indicated genotypes. Values are reported as mean ± SEM; *n* = 5 to 7 per group. *p* using Student's two-tailed *t*-test. In (h), gels were simultaneously run under same experimental conditions.

We have recently reported the central role of Nrf2 signaling pathway in the pathogenesis of DMD [11]. To better define the Nrf2-dependent antioxidant response in the prenecrotic dystrophic muscle, we analysed the expression of Nrf2 antioxidant enzymes in 2-week-old mdx mice (Figure 2). We found that the levels of several Nrf2-regulated gene expressions including SOD1/2, CAT-1, Gpx1, and GCL [25] were expressed at similar levels in both 2-week-old wild-type and mdx mice, whereas NAD(P)H quinone dehydrogenase 1 (NQO1), the enzyme catalyzing the reduction of quinones to hydroquinones, was reduced in mdx mice compared to wild-type littermates (Figure 2(a)). Of note, ROS-detoxifying enzymes, such as catalase-1 and Gpx1, were significantly reduced in mdx/IL6 compared to mdx mice, being consistent with the reduction in Nrf2 protein level (Figure 1(c)).

Interestingly, HO-1, another Nrf2-regulated gene and a modulator of the inflammatory response, was upregulated in both mdx and mdx/IL6 mice compared to wild-type littermates, although the expression of HO-1 resulted reduced in mdx/IL6 mice compared to mdx littermates (Figure 2(b)). It is plausible that the initial increase in IL-6 plasma levels induces an alteration in the redox signaling, which might stimulate the inflammatory response. To support this hypothesis, we analysed the expression of both proinflammatory cytokines such as IL-6, TNF*α*, and IL-1*β* and anti-inflammatory cytokine, such as IL-10, in 2-week-old dystrophic mice (Figures 2(c), 2(d), 2(e), and 2(f)). We found that increased plasma levels of IL-6 induced an upregulation of relevant markers of muscle wasting, such as IL6R*α* (Figure 2(g)), IL-1*β* (Figure 2(e)), TNF*α* (Figure 2(d)), and NF*κ*B (Figure 2(h)) [26, 27] in mdx/IL6 mice, compared to mdx littermates. This suggests that IL-6 triggers an alteration in the redox signaling, initiating degenerative process [22, 26, 27].

### 3.3. X-ROS Signaling Is Altered in Prenecrotic Dystrophic Diaphragm Muscle and Increases during the Progression of Pathology

In DMD, the primary defect leads to the alteration of microtubule network (MT network) that activates robust NOX2-dependent ROS production, a pathway called X-ROS [28]. A complete transcriptome analysis on biopsies of DMD patients has also revealed an upregulation of several X-ROS-related transcripts, including NOX2 and nine different tubulin isoforms [28]. Moreover, genetic silencing of Nrf2 enhances X-ROS signaling in a mild mouse model of DMD [29], suggesting a correlation between X-ROS signaling and Nrf2-dependent antioxidant response. The upregulation of NOX2's catalytic (gp91phox) subunit, which we observed in the prenecrotic dystrophic muscle (Figure 1(b)), suggests the presence of pro-oxidant conditions that could be dependent by the alteration of X-ROS signaling.

**Figure 3:**
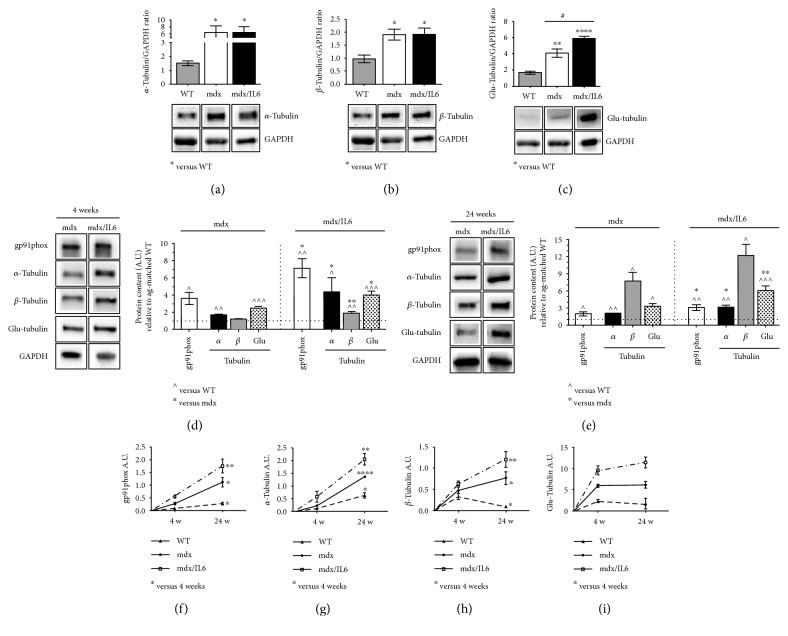
X-ROS signaling is altered in the prenecrotic dystrophic diaphragm muscle and increased during the progression of pathology. Densitometric analysis (upper panels) and representative images (bottom panels) of western blot analysis for the expression of *α*-tubulin (a), *β*-tubulin (b), and Glu-tubulin (c) proteins in the diaphragm muscle of 2-week-old WT, mdx, and mdx/IL6 mice. Values represent mean ± SEM; *n* = 3 to 7 mice per group. *p* value by ANOVA. X-ROS signaling components were analysed by western blot (left panels show representative images) at later stages of pathology in 4-week-old (d) and 24-week-old (e) diaphragm muscle from WT, mdx, and mdx/IL6 mice. Values are reported as protein content relative to age-matched WT (horizontal dot line) and represent mean ± SEM; *n* = 3 to 7 mice per group. ^^^*p* < 0.05, ^^^^*p* < 0.005, and ^^^^^*p* < 0.0005 compared to WT mice; ^∗^*p* < 0.05, ^∗∗^*p* < 0.005 with respect to mdx littermates (by ANOVA). Temporal progression of gp91phox (f), *α*-tubulin (g), *β*-tubulin (h), and Glu-tubulin (i) proteins between 4 weeks (4 w) and 24 weeks (24 w) of age in the diaphragm muscles of indicated genotypes. Graphs show an increase of the expression levels of tubulin subunits and of gp91phox protein during the progression of pathology. Values represent mean ± SEM. ^∗^*p* < 0.05, ^∗∗^*p* < 0.005, and ^∗∗∗∗^*p* < 0.0001 by ANOVA.

To support this hypothesis, we analysed the density of MT network in terms of the abundance of *α*-, *β*-, and Glu-tubulin proteins, in dystrophic muscles at 2 weeks of age (Figures 3(a), 3(b), and 3(c)). We found an upregulation of the overall subunits in mdx and mdx/IL6 compared to wild type mice, indicating an alteration of microtubule network at this stage of pathology. Notably, we observed a significant increase in detyrosination of tubulin content (Glu-tubulin), but not of the *α* (Figure 3(a))- and *β* (Figure 3(b))-tubulin subunits, in the diaphragm of mdx/IL6 mice compared to mdx littermates (Figure 3(c)).

To verify whether ROS production parallels X-ROS signaling during the progression of DMD pathology, we analysed gp91phox expression and microtubule subunits' content in dystrophic mice at 4 and 24 weeks of age (Figures 3(d) and 3(e)). We found that the dystrophic muscle displayed per se increased levels of gp91phox and tubulin subunit expression at both 4 (Figure 3(d)) and 24 weeks of age (Figure 3(e)) compared to wild type, whereas increased levels of IL-6 significantly enhanced content of gp91phox and of *α*-, *β*-, and Glu-tubulin proteins compared to mdx mice, suggesting that IL-6 exacerbates the dystrophic phenotype acting also on the stimulation of X-ROS signaling.

### 3.4. Increased Levels of IL-6 Affect Nrf2 Antioxidant Response during the Progression of Pathology

**Figure 4:**
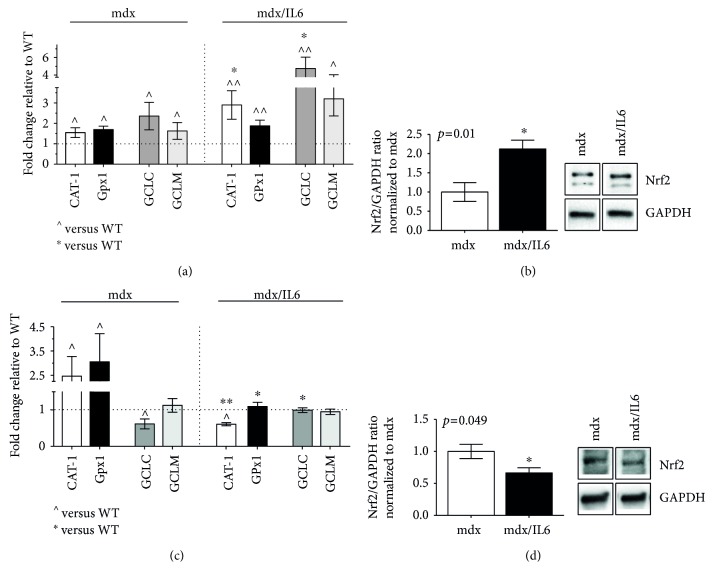
Time course analysis of Nrf2-dependent antioxidant response in dystrophic mice. Real-time PCR analysis performed on diaphragm muscles from wild-type (WT), mdx, and mdx/IL6 mice at 4 (a) and 24 (c) weeks of age for the expression of Nrf2-dependent genes involved in the antioxidant response. Values are reported as fold change in the expression relative to the calibrator (WT, horizontal dot line) and represent mean ± SEM; *n* = 3 to 6 mice per group. ^^^*p* < 0.05, ^^^^*p* < 0.005 compared to WT mice; ^∗^*p* < 0.05, ^∗∗^*p* < 0.005 with respect to mdx littermates (by ANOVA). Nrf2 protein expression was evaluated by western blot analysis (right panels show representative images) in 4-week-old (b) and 24-week-old (d) diaphragms of indicated genotypes. Densitometric analyses (left panels) are expressed as values relative to age-matched mdx and represent mean ± SEM; *n* = 4 to 5 mice per group. *p* value using Student's two-tailed *t*-test.

In order to evaluate the correlation between the temporal progression of X-ROS signaling and Nrf2-dependent antioxidant response in muscular dystrophy and to better define the pathogenic role of IL-6 in muscular dystrophy, we analysed the Nrf2-mediated antioxidant enzyme expression in the diaphragm of mdx and mdx/IL6 mice at different stages of pathology (Figure 4). In particular, we evaluated the antioxidant response in dystrophic mice at two different ages and stages of disease, namely, at 4 weeks of age, in which a peak of necrosis is observed, and at 24 weeks of age, a stage in which the affected muscle rapidly regenerates and regains structural and functional integrity [2].

We found that most of the antioxidant enzymes, including CAT-1, Gpx1, and GCL, were upregulated in 4-week-old mdx and mdx/IL6 mice compared to wild-type littermates (Figure 4(a)). Notably, the increased plasma levels of IL-6 induced a more significant upregulation of CAT-1 compared to mdx mice (Figure 4(a)). We also found that the levels of mRNAs that encode the components of gamma-glutamyl-cysteine ligase and the rate-limiting enzyme for glutathione biosynthesis (glutamyl-cysteine ligase modulator (GCLM) and glutamyl-cysteine ligase catalytic subunit (GCLC)) were expressed at higher levels in mdx/IL6 mice compared to mdx littermates, indicating that IL-6 overexpression induces a dysregulation of GSH synthesis in dystrophic muscles. These data suggest that the exacerbated muscle phenotype, induced by increased levels of IL-6 is associated with an imbalance between oxidant and antioxidant systems, as also suggested by increased expression of Nrf2 protein in 4-week-old mdx/IL6 mice compared to mdx littermates (Figure 4(b)).

Then, we analysed the expression pattern of the antioxidant enzymes in both mdx and mdx/IL6 mice of 24 weeks of age, a stage normally spared by the absence of dystrophin. We observed that CAT-1 and Gpx1, which catalyze the conversion of hydrogen peroxide to water and oxygen, were still upregulated in the diaphragm of 24-week-old mdx mice compared to both wild-type and mdx/IL6 mice (Figure 4(c)). This suggests that the antioxidant compensatory mechanism is still activated in adult mdx muscles and this might explain the mild muscle phenotype observed in mdx mice at this stage of pathology. In contrast, increased levels of IL-6 induced a significant downregulation of the ROS-detoxifying enzymes, compared to mdx mice, suggesting that IL-6 overexpression negatively affects the compensatory response in the dystrophic muscle. This evidence was supported by the significant reduction of Nrf2 protein expression in 24-week-old mdx/IL6 mice compared to mdx littermates (Figure 4(d)).

## 4. Discussion

In this study, we monitored relevant markers of the redox signaling in the dystrophic muscle and suggested a potential link between increased circulating levels of IL-6 and oxidative damage.

Duchenne muscular dystrophy is an X-linked genetic disease due to mutations in the dystrophin gene, leading to alterations in intracellular signaling that causes an imbalance between protein synthesis and protein degradation, with subsequent necrosis and fibrosis [1].

Currently, there is no effective therapy for Duchenne muscular dystrophy. Although stem cells and exon skipping approach offer new tools for regeneration in muscle disease, the signaling and molecular pathway involved in the survival of the rescued phenotype is an important question that remains to be satisfactorily addressed. Among factors that might interfere with therapeutic approaches, the dystrophic environment represents an important determinant [30]. Thus, a better understanding of the hostile microenvironment should prove useful for producing new adjuvant treatments.

Potential candidates that contribute to sustain a hostile microenvironment in the dystrophic muscle include oxidative stress [4] and interleukin-6 (IL-6), a pleiotropic cytokine that is produced by different cell types and has the capacity to induce several different intracellular signaling pathways [3, 10, 31]. The skeletal muscle is able to activate, under physiological conditions, an endogenous program of antioxidant defense to maintain the ROS production at a functional level [7]. Nevertheless, damaging stimuli might alter the delicate balance between ROS production and antioxidant defense, leading to oxidative stress. The opposite effects exerted by different concentration of ROS can be justified considering the concept of hormesis [32], in which a low dose of a substance is stimulatory and a high dose is inhibitory. Thus, the muscle benefits from low doses of radicals while it is damaged by higher levels of ROS.

Our work supports the evidence that dystrophic muscle is able to activate a compensatory response to cope the negative effects of oxidative stress. Nevertheless, this compensatory mechanism is impinged by factors that are associated with the pathogenesis of muscular dystrophy, such as IL-6. Indeed, the persistent activation of IL-6 signaling impairs the antioxidant response during the progression of pathology, contributing to the severity of pathology.

In particular, we first verified whether relevant markers of the redox signaling are direct targets of necrosis and inflammation, which characterize the necrotic stage of the disease, or whether they can be induced in the prenecrotic stage. We found a strong upregulation of one of the relevant and critical factors for ROS production in the dystrophic muscle, namely, gp91phox (NOX2), and a significant reduction in SIRT1 and Nrf2 expression, important mediators of the antioxidant response, in the diaphragm muscle of 2-week-old mdx mice compared to wild-type littermates. This indicates that the activation of redox-regulated signaling precedes necrosis rather than resulting from it and supports oxidative stress as a primary pathogenetic mechanism in muscular dystrophy [4].

We also analysed the relevant markers of the X-ROS signaling at 4 weeks (necrotic stage) and 24 weeks (a stage normally spared by the absence of dystrophin) of age in mdx mice. We revealed that X-ROS signaling is already altered in the dystrophic diaphragm at the necrotic stage, whereas markers of the antioxidant compensatory mechanism were upregulated in adult mdx muscles.

To further support the evidence that the imbalance between oxidant and antioxidant mechanisms is a pathogenic factor associated with the severity of pathology, we analysed the markers of X-ROS signaling in the diaphragm muscle of mdx/IL6 mouse model, which recapitulates the severe phenotypic characteristics of DMD in humans [3, 10]. Sustained increase in the levels of IL-6 alone is sufficient to exacerbate the dystrophic phenotype at a stage, 24 weeks of age, when only a mild muscle phenotype is apparent in mdx mice [10]. We revealed that increased levels of IL-6 induced a significant downregulation of the ROS-detoxifying enzymes, compared to mdx mice, and negatively affect the compensatory response in the dystrophic muscle.

## 5. Conclusion

**Figure 5:**
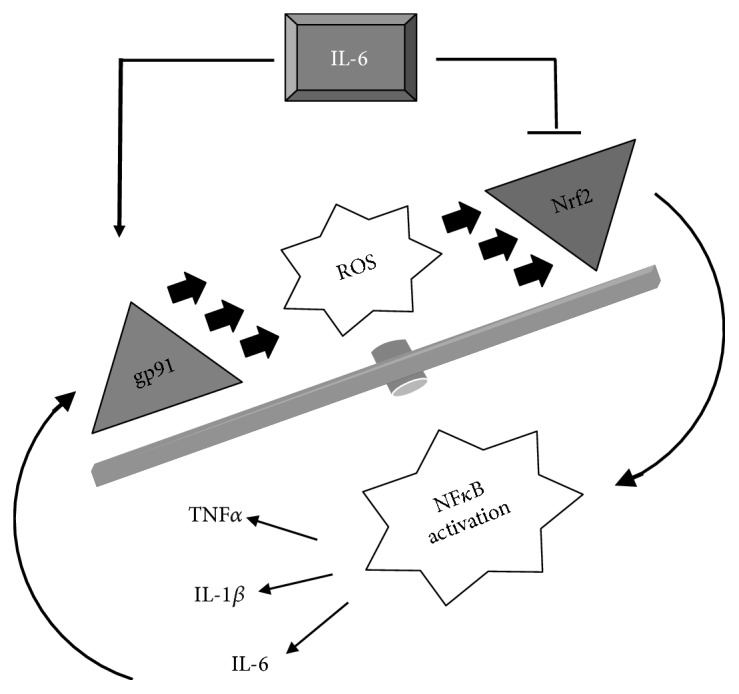
IL-6 exacerbates the oxidant-related damage in the dystrophic muscle. Increased levels of IL-6 could contribute to amplify degenerative processes in mdx mice by enhancing the expression of both gp91phox and NF*κ*B and by reducing the Nrf2-antioxidant response.

Overall, our study is consistent with the model in which IL-6 could act as an important player in the crosstalk between ROS production and inflammatory response [22]. Increased levels of IL-6 enhance the expression of both gp91phox and NF*κ*B and impinge the Nrf2-dependent antioxidant response in the dystrophic muscle, even at the prenecrotic stage (Figure 5). This altered redox-regulated signaling might trigger an inflammatory response, leading to muscle wasting.

## Abbreviations

ANOVA: Analysis of variance
CAT-1: Catalase-1
DHE: Dihydroethidium
DMD: Duchenne muscular dystrophy
DTT: Dithiothreitol
EDTA: Ethylenediaminetetraacetic acid
EGTA: Ethylene glycol tetraacetic acid
GAPDH: Glyceraldehyde 3-phosphate dehydrogenase
GCL: Glutamate-cysteine ligase
GSH: Glutathione
GPx: Glutathione peroxidase
HO-1: Heme oxygenase 1
Hprt: Hypoxanthine guanine phosphoribosyl transferase
IL: Interleukin
IL-6R*α:*: Interleukin-6 receptor alpha
NaF: Sodium fluoride
NF*κ*B: Nuclear factor kappa-light-chain-enhancer of activated B cells
NOX2: NAPDH oxidase 2
Nrf2: NF-E2-related factor 2
NQO1: NAD(P)H quinone dehydrogenase 1
PMSF: Phenylmethylsulfonyl fluoride
ROS: Reactive oxygen species
SEM: Standard error of the mean
SIRT1: Sirtuin 1
SOD: Superoxide dismutase
SOV4: Sodium orthovanadate
TNF: Tumor necrosis factor
Utrn: Utrophin.
